# Supplementary material for: Content-rich biological network constructed by mining PubMed abstracts
Source: BMC Bioinformatics. 2004 Oct 8;5:147. doi: 10.1186/1471-2105-5-147 (PMC528731; doi:10.1186/1471-2105-5-147)
Supplement: Additional File 2 — The original results of the above study (non-essential files are deleted to keep the file size under the limit set by BMC bioinformatics). [file 1471-2105-5-147-S2.bz2 › chilibotAdditionalFile2/dip30/360ID9195914E5748/html/IL8_IL8RB.html]

 


 **IL8** and **IL8RB** 
  
Found 192 abstracts in PubMed, retrieved 30.  
 

 What does Google say? 
 PDF only 
| .edu only 

---

**Interactive relationship** (e.g. stimulation, inhibition, etc)

**Neutral relationship**- IL 8  [ **IL8** ]  Induced Migratory Responses through CXCR1 and CXCR2  [ **IL8RB** ]  Association with Phosphorylation and Cellular Redistribution of Focal Adhesion Kinase.  Ref: 12627953 Biochemistry, 2003
- The migration activating concentrations of 50 ng mL GCP  [ **IL8** ]  2 and NAP  [ **IL8** ]  2 induced less potent migratory responses and FAK phosphorylation in CXCR2  [ **IL8RB** ]  expressing cells as compared with IL 8  [ **IL8** ] .  Ref: 12627953 Biochemistry, 2003
- Special emphasis is placed on the interaction of the human immunodeficiency virus HIV Tat protein with endothelial, astroglial, microglial, and neuronal cells, resulting in altered expression of vascular endothelial growth factor, fibroblast growth factor, interleukin 8  [ **IL8** ] , and regulation of calcium flux via CXCR2  [ **IL8RB** ] , which directly influences neuronal cell fitness.  Ref: 12476355 J Neurovirol, 2002
- Characterization of the molecular interactions of interleukin 8  [ **IL8** ]  CXCL8  [ **IL8** ] , growth related oncogen alpha CXCL1 and a non peptide antagonist SB 225002 with the human CXCR2  [ **IL8RB** ] .  Ref: 12628493 Biochem Pharmacol, 2003
- Here, we show that survival of cultured astrocytes after Fas triggering is governed by the interaction of interleukin 8  [ **IL8** ]  with one of its receptors, CXCR2  [ **IL8RB** ] .  Ref: 12395092 Neuroreport, 2002
- Angiogenic effects of interleukin 8  [ **IL8** ]  CXCL8  [ **IL8** ]  in human intestinal microvascular endothelial cells are mediated by CXCR2  [ **IL8RB** ] .  Ref: 12496258 J Biol Chem, 2003
- CXCR1 and CXCR2  [ **IL8RB** ]  mediate migratory activities in response to IL 8  [ **IL8** ]  and other ELR CXC chemokines e.g., GCP  [ **IL8** ]  2 and NAP  [ **IL8** ]  2.  Ref: 12627953 Biochemistry, 2003
- IL 8  [ **IL8** ]  or CXCL8  [ **IL8** ]  activates the receptors CXCR1 IL 8RA and CXCR2  [ **IL8RB** ]  IL 8RB to induce chemotaxis in leukocytes, but only CXCR1 mediates cytotoxic and cross regulatory signals.  Ref: 12626541 J Immunol, 2003
- IL 8  [ **IL8** ]  elicits angiogenic responses in microvascular endothelial cells isolated from human intestine by engaging CXCR2  [ **IL8RB** ] .  Ref: 12496258 J Biol Chem, 2003
- CXCL8  [ **IL8** ]  exerts potent angiogenic properties on endothelial cells through interaction with its cognate receptors CXCR1 and CXCR2  [ **IL8RB** ] .  Ref: 12496258 J Biol Chem, 2003
- The effects of IL 8  [ **IL8** ]  and its related ligands are mediated via two receptors, CXCR1 and CXCR2  [ **IL8RB** ] .  Ref: 12444152 J Immunol, 2002
- The CXCR2  [ **IL8RB** ]  is a GPCR that is activated, amongst the others, by the chemokines CXCL8  [ **IL8** ]  IL 8  [ **IL8** ]  and CXCL2 GROb to induce cell chemotaxis.  Ref: 12888558 J Biol ChemJ Biol Chem, 2002
- We recently engineered a series of high affinity CXCL8  [ **IL8** ]  IL 8  [ **IL8** ]  antagonists, one of which, CXCL8  [ **IL8** ]  3 73 K11R G31P, binds very strongly to neutrophils via the CXCR1 and CXCR2  [ **IL8RB** ] .  Ref: 12406656 Vet Immunol Immunopathol, 2002
- As CXCR1 and CXCR2  [ **IL8RB** ]  expression is differentially regulated in tissue specific endothelial cells and effects of IL 8  [ **IL8** ]  on intestinal endothelial cells are not defined, we characterized the potential IL 8  [ **IL8** ]  induced angiogenic mechanisms in primary cultures of human intestinal microvascular endothelial cells HIMEC and IL 8  [ **IL8** ]  receptor expression in human intestinal microvessels.  Ref: 12496258 J Biol Chem, 2003
- The induction of IL 8  [ **IL8** ]  in HAD, its inhibition of LTP, and the expression of its receptor, CXCR2  [ **IL8RB** ] , in the hippocampus all suggest that it plays a role in the cognitive dysfunction associated with HAD.  Ref: 12548717 J Neurosci Res, 2003

**Non-interactive relationship** (e.g. studied together, co-existance, homology, etc.)

- Thus in COPD, in addition to CXCL8  [ **IL8** ]  and CXCR1, CXCL5 and CXCR2  [ **IL8RB** ]  appear to play important roles in the airway neutrophilia characteristic of severe exacerbations.  Ref: 12857718 Am J Respir Crit Care MedAm J Respir Crit Care Med, 2003
- However, the contribution of polymorphisms of the IL 8  [ **IL8** ]  gene and its receptor genes CXCR 1 and CXCR 2  [ **IL8RB** ]  to human TB susceptibility remains untested.  Ref: 12870115 J Infect Dis, 2003
- A self defence mechanism of astrocytes against Fas mediated death involving interleukin 8  [ **IL8** ]  and CXCR2  [ **IL8RB** ] .  Ref: 12395092 Neuroreport, 2002
- Neutralizing antibodies to CXCR2  [ **IL8RB** ]  diminished IL 8  [ **IL8** ]  induced chemotaxis and stress fiber assembly.  Ref: 12496258 J Biol Chem, 2003
- In this study, we addressed the question of whether expression of the interleukin 8  [ **IL8** ]  receptors IL 8RA CXCR1 and IL 8RB CXCR2  [ **IL8RB** ]  is upregulated in H. pylori infected human gastric biopsy samples.  Ref: 12761119 Infect Immun, 2003
- IL 8  [ **IL8** ]  activation of CXCR1, but not the IL 8  [ **IL8** ]  receptor CXCR2  [ **IL8RB** ] , cross phosphorylated CCR5 and CXCR4 and cross desensitized their responsiveness to RANTES regulated on activation normal T cell expressed and secreted CCL5 and stromal derived factor SDF 1 or CXCL12, respectively.  Ref: 12594210 J Biol Chem, 2003
- Our data suggest that IL 8  [ **IL8** ]  directly enhanced endothelial cell proliferation, survival, and MMP expression in CXCR1 and CXCR2  [ **IL8RB** ]  expressing endothelial cells and regulated angiogenesis.  Ref: 12626597 J Immunol, 2003
- In the present study, we demonstrate that a potent and selective nonpeptide antagonist of human CXCR2  [ **IL8RB** ]  potently inhibits 125 I labeled human IL 8  [ **IL8** ]  binding to, and human IL 8  [ **IL8** ]  induced calcium mobilization mediated by, rabbit CXCR2  [ **IL8RB** ]  IC 50 = 40.5 and 7.7 nM, respectively, but not rabbit CXCR1 IC 50 = >1000 and 2200 nM, respectively.  Ref: 12444152 J Immunol, 2002
- Both IL 8  [ **IL8** ]  receptors, CXCR1 and CXCR2  [ **IL8RB** ] , are expressed extensively in the fetal intestine.  Ref: 12633567 Cytokine, 2002
- The immunohistochemical analysis using specific antibodies demonstrated that positive staining for IL 8  [ **IL8** ] , CXCR1 and CXCR2  [ **IL8RB** ]  in surgically resected human pancreatic cancer was 5 5 and 65%, respectively.  Ref: 12632066 Int J Oncol, 2003
- Analysis of inter relationships between CXCR1 and CXCR2  [ **IL8RB** ]  during internalization indicated that the exposure of cells that expressed both CXCR1 and CXCR2  [ **IL8RB** ]  to CXCL8  [ **IL8** ]  or CXCL6 resulted in decreased levels of CXCR1 internalization as compared to those in cells that expressed only CXCR1.  Ref: 12442335 Eur J Immunol, 2002
- By comparison with the NSC and S COPD groups, neutrophilia and gene expression for epithelial derived neutrophil attractant 78 CXCL5, interleukin 8  [ **IL8** ]  CXCL8  [ **IL8** ] , CXCR1 and CXCR2  [ **IL8RB** ]  were each up regulated in the E COPD group p&lt.  Ref: 12857718 Am J Respir Crit Care MedAm J Respir Crit Care Med, 2002
- Furthermore, interleukin 8  [ **IL8** ]  secretion and CXCR2  [ **IL8RB** ]  expression are both induced in human astrocytes after Fas stimulation, suggesting a new mechanism of self defence against Fas mediated death.  Ref: 12395092 Neuroreport, 2002
- In contrast, in CXCR2  [ **IL8RB** ]  RBL cells the migration attenuating concentrations of IL 8  [ **IL8** ]  induced promoted levels of FAK phosphorylation and different patterns of FAK phosphorylation on its six potential tyrosine phosphorylation sites, as compared to activating concentrations of the chemokine.  Ref: 12627953 Biochemistry, 2003
- OBJECTIVE We sought to investigate the in vivo effect of bradykinin on the expression of the IL 8  [ **IL8** ]  receptors CXCR1 and CXCR2  [ **IL8RB** ]  in nasal cells.  Ref: 12532104 J Allergy Clin Immunol, 2003
- We examined the expression of IL 8  [ **IL8** ]  and its two receptors CXCR1 and CXCR2  [ **IL8RB** ]  in 40 surgically resected human pancreatic cancer tissues and in three different human pancreatic cancer cell lines PANC 1, MIAPaCa 2 and Capan 2.  Ref: 12632066 Int J Oncol, 2003
- The stimulation of CXCR1 and CXCR2  [ **IL8RB** ]  by IL 8  [ **IL8** ]  concentrations that result in migratory activation induced focal adhesion kinase FAK phosphorylation in a G alpha i dependent manner.  Ref: 12627953 Biochemistry, 2003
- Finally, the mechanism of binding of a non peptide antagonist on CXCR2  [ **IL8RB** ]  was investigated it occurred through epitopes a which were disseminated within the receptor, b which differed according to the use of CXCL8  [ **IL8** ]  or CXCL1 as a competitor and c which did not necessarily overlap with agonist binding sites.  Ref: 12628493 Biochem Pharmacol, 2003
- Preincubation of hippocampal slices with a monoclonal CXCR2  [ **IL8RB** ]  antibody 2 microg ml but not control IgG 2 microg ml blocked IL 8  [ **IL8** ]  induced inhibition of LTP.  Ref: 12548717 J Neurosci Res, 2003
- The presence of wortmannin during receptor recycling inhibited CXCR1 and CXCR2  [ **IL8RB** ]  re expression following CXCL8  [ **IL8** ]  induced internalization, and resulted in a marked disruption of the proper organization of actin filaments.  Ref: 12442335 Eur J Immunol, 2002
- Supported by the notion that malignant colonic epithelial cells overexpress IL 8  [ **IL8** ] , CXCR2  [ **IL8RB** ]  blockade may be a novel target for anti angiogenic therapy in colorectal adenocarcinoma.  Ref: 12496258 J Biol Chem, 2003
- The mutants generated showed that a for CXCL8  [ **IL8** ]  binding, the N terminus of CXCR1 and the second extra cellular loop of CXCR2  [ **IL8RB** ]  are determinant, the N terminus of CXCR2  [ **IL8RB** ]  is not sufficient and the transmembrane domain seven is probably involved.  Ref: 12628493 Biochem Pharmacol, 2003
- In the present study, we examined the expression of chemokine receptors CXCR1 and CXCR2  [ **IL8RB** ]  receptors for interleukin 8  [ **IL8** ]  and CCR5 receptor for RANTES regulated on activation, normal T cell expressed and secreted, macrophage inflammatory protein MIP 1alpha, and MIP 1beta in human endometrium.  Ref: 12606476 Biol Reprod, 2003
- There are at least two different IL 8  [ **IL8** ]  receptor types CXCR1 and CXCR2  [ **IL8RB** ] .  Ref: 12561382 Sheng Wu Yi Xue Gong Cheng Xue Za Zhi, 2002
- It is well known that chemokines and chemokine receptors, such as interleukin 8  [ **IL8** ]  and its receptors CXCR1 and CXCR2  [ **IL8RB** ] , play important roles in the pathogenesis of psoriasis.  Ref: 12823305 Clin Exp Dermatol, 2003
- In vitro, the antagonist potently inhibited human IL 8  [ **IL8** ]  induced chemotaxis of rabbit neutrophils IC 50 = 0.75 nM, suggesting that inhibition of leukocyte migration into the knee joint is a likely mechanism by which the CXCR2  [ **IL8RB** ]  antagonist modulates disease.  Ref: 12444152 J Immunol, 2002
- Expression of the chemokine receptors CCR2, specific for MCP 1, and CXCR1 and CXCR2  [ **IL8RB** ] , specific for IL 8  [ **IL8** ] , were not altered by HCMV infection.  Ref: 12714640 Invest Ophthalmol Vis Sci, 2003
